# Clinical metagenomic sequencing for rapid diagnosis of neonatal meningitis caused by *Ureaplasma parvum*: A case report

**DOI:** 10.1097/MD.0000000000028662

**Published:** 2022-01-28

**Authors:** Lyn Qin, Yan-hong Li, Xue-jie Cao, Xiao-jun Wang, Ren-ping Mao, Hai-yin Yang, Li Li

**Affiliations:** a Neonatal Intensive Care Unit, Ningbo Women and Children's Hospital 339 Liuting Street, Haishu District, Ningbo, China; b Genoxor Medical Science and Technology Inc., Shanghai, China.

**Keywords:** mNGS, neonatal meningitis, *Ureaplasma parvum*

## Abstract

**Introduction:**

It is challenging to obtain favorable results through conventional diagnostic testing for *Ureaplasma parvum* (UP), a conditional pathogen, because of the atypical clinical phenotype of UP meningitis.

**Patient concerns and diagnosis:**

Herein, we report a pediatric case of neonatal meningitis caused by UP in a spontaneously delivered full-term baby. The infant's temperature peak was 38.3°C at the age of 9 days. The patient was diagnosed with neonatal suppurative meningitis.

**Interventions and outcomes:**

The pathogen was diagnosed in a timely and accurate manner by metagenome sequencing, and the patient was eventually discharged with azithromycin.

**Conclusions:**

Neonatal Ureaplasma meningitis may be more common than previously suspected. The clinical manifestations were not obvious and were similar to those of neonatal meningitis caused by other bacteria. When conventional treatments and conventional pathogenic tests are negative, mNGS is a better choice for timely and accurate pathogen identification.

## 1 Introduction

*Ureaplasma parvum* (UP) belongs to the Ureaplasma genus. It is the smallest prokaryotic microbe among bacteria and viruses and is capable of self-replication. It often resides in the urogenital tract as a causative pathogen. Ureaplasma is a bacterial genus with a high rate of cervical infection in women, and the symptoms of infection are not apparent.^[1]^ Infection can cause vaginitis, cervicitis, salpingitis, pelvic inflammatory disease, infertility, and miscarriage in women. In some cases, it can lead to central nervous system infections in premature and full-term newborns and cause neonatal meningitis.^[2]^ It is difficult to obtain favorable results through conventional diagnostic testing because of the atypical clinical phenotype of UP meningitis. Unlike traditional detection methods, metagenomic next-generation sequencing (mNGS) can detect tens of thousands of microorganisms simultaneously and is increasingly used in clinics. Here, we describe the case of a natural full-term male baby who was diagnosed with neonatal meningitis. However, the conventional diagnostic method is invalid. The pathogen was diagnosed as UP using mNGS and confirmed by PCR at the same time. This rare case was successfully treated and the patient was discharged after treatment with azithromycin.

## 2 Case presentation

The patient was a male with a G3P1A2 gestational age of 39 + 6 weeks. He delivered naturally through the vagina and had a birth weight of 3250 g. The results of routine blood glucose testing after birth showed that “blood sugar was high for more than 1 hour.” Initial diagnosis:

1. neonatal purulent meningitis
2. neonatal sepsis
3. neonatal hyperglycemia
4. neonatal pneumonia
5. anemia
6. myocardial damage
7. neonatal hyperbilirubinemia.

**Figure 1:**
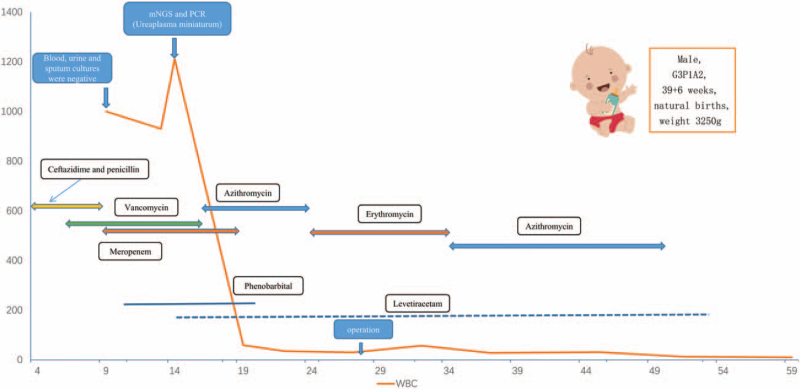
Molecular detection technology-qPCR. Real-time qPCR amplification curve confirms *Ureaplasma parvum.* The red and yellow amplification curve represents the result of this sample, the green amplification curve represents the positive control, and the blue amplification curve represents the negative control.

**Figure 2:**
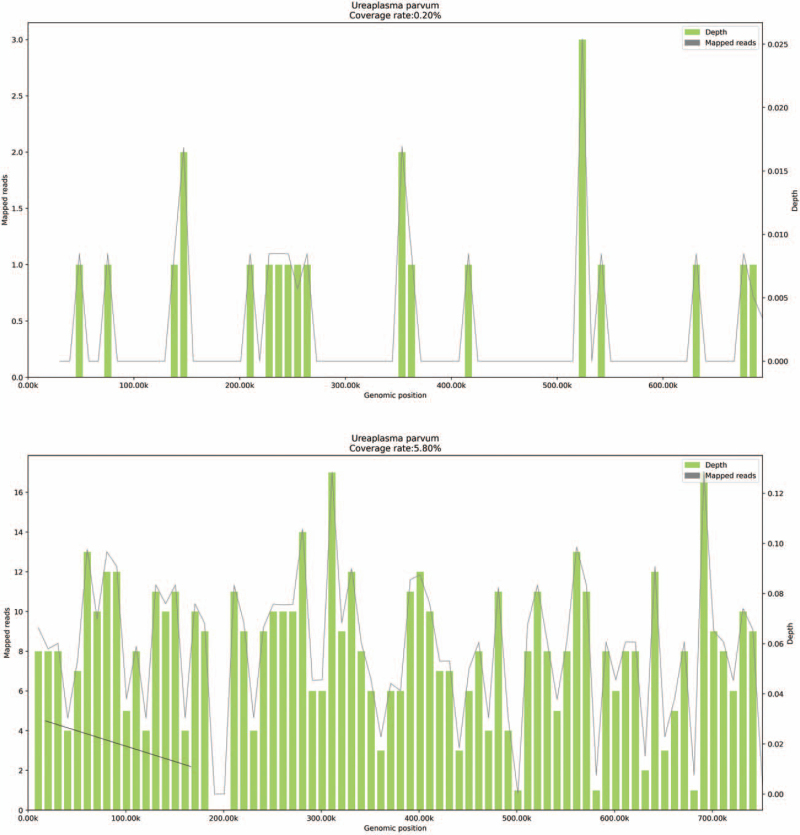
Molecular detection technology-mNGS. Genome coverage of *Ureaplasma parvum.*

He was administered ceftazidime combined with penicillin intravenously and symptomatic support, such as oxygen inhalation and dopamine, to improve circulation. The infant's heat peak reached 38.3°C at the age of 9 days, and the local hospital's cerebrospinal fluid routine: WBC 1000/μL, RBC 6000/μL, monocytes 84%, glucose 2.08 mmol/L. The patient was diagnosed with neonatal suppurative meningitis. As his condition changed rapidly, he was transferred to our hospital for further treatment. There was no vomiting, convulsions, cyanosis, or apnea during the day. Blood routine: WBC 16.9 × 109, PCT 0.209 ng/dL, CRP 38.6 mg/L. Blood, cerebrospinal fluid, urine culture, and microscopic examination Thirteen joint inspections of the respiratory tract for pathogenic testing. The next day, head B-ultrasonography showed a slightly strong echo (41 × 28 × 36) in the brain parenchyma on the left and subdural and fusiform echoes of the left temporal roof, with a range of approximately 23 × 4 × 15. Ultrasound showed that the right lateral ventricle was dilated with a slightly enhanced echo of the right choroid plexus. The left brain parenchyma with a somewhat stronger echo was considered to be bleeding, and the left temporal top subdural without echo was considered to be a subdural hematoma. Pathogen detection results were negative; meropenem and vancomycin were used for empirical treatment, and inflammation indicators were monitored during this period. After 5 days of treatment, the symptoms were not alleviated, and the infant continued to have a fever. The infant's CSF was sent for mNGS testing. The reported UP, supported by 12 sequences within 36 hours, was further verified by qPCR (Fig. 1). The specific PCR analysis was positive, and the amplification primers were 5’-CATTGATGTTGCACAAGGAG-3’ and 5’-CGTGATTTTAATGTATCGGCTTTTC-3’. Subsequently, the patient was diagnosed with Ureaplasma micromeningitis, vancomycin and meropenem were discontinued, azithromycin (dose) treatment was increased, and erythromycin injection after 7 days. After one week, the number of leukocytes in the CSF increased, and the glucose concentration in the CSF decreased. Considering the low permeability of the erythromycin blood-brain barrier, azithromycin was adjusted to continue treatment. The infant was performed “left temporal craniotomy hematoma removal” under general anesthesia at 24 days. mNGS analysis of intraoperative pathogenic microorganisms of purulent adherents suggested the presence of UP (Fig. 2).

**Figure 3:**
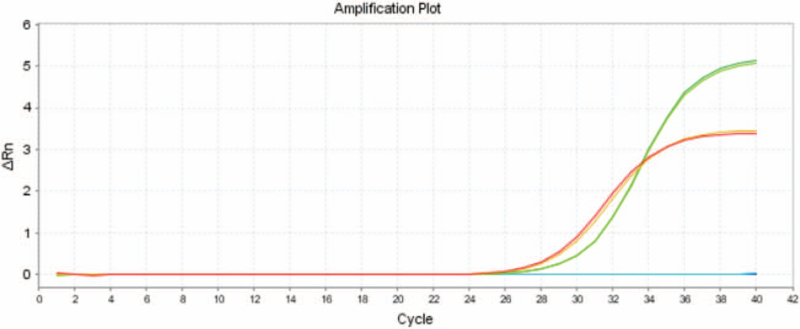
White blood cell count in cerebrospinal fluid, medication regimen, and etiological results. The horizontal line represents medication and medication cycles. The ordinate is the value of cerebrospinal fluid white blood cells and the abscissa represents age.

In addition to head B-ultrasound monitoring, anti-infective treatment was continued postoperatively. After 5 weeks of treatment, the cerebrospinal fluid test recovered, the condition was stable, and the infant was discharged from the hospital. Follow-up was continued and no abnormalities were observed. The specific treatment and CSF examination of the infant are shown in Figure 3.

## 3 Discussion and conclusion

This article describes a case of neonatal meningitis caused by UP. Routine tests of infant blood, cerebrospinal fluid, and urine yielded negative results. mNGS detected UP, which was further confirmed by PCR. After adjusting for erythromycin combined with azithromycin anti-infective treatment, the infant was successfully discharged.

Ureaplasma is the smallest prokaryotic microorganism with no cell wall and high pleomorphism, which can be artificially cultured and increased through a bacterial filter. They mainly exist in the human urinary tract, reproductive tract, and respiratory tract and are common pathogens that cause urogenital infections.^[3]^ Ureaplasma can cause adverse pregnancy outcomes in premature infants and infected infants. A previous report showed that a premature infant born at less than 32 weeks of gestation had Ureaplasma detected in multiple locations (respiration, blood, or cerebrospinal fluid).^[4]^ In a prospective study of 100 cases of meningitis in premature infants, Ureaplasma and Mycoplasma hominis were frequently isolated.^[5]^ At present, only 6 cases of neonatal meningitis with UP have been reported (Table 1).^[6]^ According to reported cases, the clinical symptoms of neonatal meningitis caused by UP mainly include fever, apnea, cardiovascular instability, rhinitis, conjunctivitis, seizures, and hydrocephalus. The clinical process may be complicated by intraventricular hemorrhage, hydrocephalus, suppurative manifestations, and cerebral infarction. Cerebrospinal fluid detection often shows leukocytosis, significantly increased protein levels, and decreased glucose levels. UP-induced neonatal meningitis lacks specific central nervous system manifestations in its early stages. Reasons such as low clinical predictability and limited pathogen detection technology may be the possibility of fewer reported cases.

**Table 1:** Basic characteristics of neonatal meningitis caused by *Ureaplasma parvum.*

Gender	Gestation(week)	Onset time after birth (day)	Test method	Symptom	Complication	Treatment	Reference
Male	30	18	PCR	Fever, epilepsy, cardiovascular instability, apnea	Subdural hemorrhage and hydrocephalus	ERY+CIP × 3 weeks	^[3]^
Unknown	39	10	PCR + Culture	Fever, epilepsy	Rhinitis, conjunctivitis, ventricular dilatation	CIP × 7 weeks + THI × 3 weeks	^[7]^
Female	26 + 3	28	PCR	Weakness and relaxation, low muscle tension and lack of tendon reflex;	Hydrocephalus and growth retardation	CHL × 3 weeks	^[11]^
Female	Full term	6	PCR	Irritability, fever, epilepsy	Ventricular dilatation, ventriculitis, subdural effusion	ERY+CIP × 5 weeks+ AZY+CIP × 1 weeks	^[8]^
Male	40	11	mNGS	Fever, relaxation, epilepsy	Hydrocephalus	ERY × 4 weeks	^[9]^
Male	40	5	mNGS	Fever, epilepsy	Subdural hemorrhage, lateral ventricle enlargement	ERY × 5 weeks	^[12]^
Male	39+6	10	mNGS + PCR	Fever, epilepsy	Subdural hemorrhage, lateral ventricle enlargement	ERY × 2 weeks+ AZI × 3 weeks	The Case

mNGS overcomes the limitations of specific pathogen detection methods It is helpful to detect pathogens with atypical clinical features or common pathogens that are clinically neglected, and to identify rare and novel pathogens.^[7]^ As far as the current report is concerned, the sensitivity of mNGS is higher than that of culture, and it is less affected by antibiotics and can produce results within 24 hours.^[8]^ It has shown good performance in the diagnosis of pathogens associated with nerve infections.^[9]^ It is recommended to use mNGS when the routine microbiological test is negative and routine treatment cannot improve the infection or when the patient is critically ill.

The treatment of neonatal invasive Ureaplasma infection is challenging. Ureaplasma lacks a cell wall and is insensitive to antibiotics that inhibit cell wall synthesis.^[10]^ Antibiotics that target protein or DNA synthesis, such as macrolides, tetracyclines, clindamycin, chloramphenicol, and fluoroquinolones, may be effective. The 6 previously reported cases were also treated with macrolide or fluoroquinolone antibiotics. According to reports, tetracyclines seem to be the most effective based on in vitro tests because tetracyclines can penetrate well into the cerebrospinal fluid. However, because tetracyclines may have adverse side effects on bone and tooth development, they should be used with extreme caution in neonatal treatment.^[11]^ According to the in vitro susceptibility test results, the effect of doxycycline penetrating the cerebrospinal fluid was better than that of erythromycin. However, among the reported cases, some were successfully treated with erythromycin alone. This may be due to the increased permeability of the blood-brain barrier during meningitis. However, some cases have reported that erythromycin alone cannot completely eradicate Ureaplasma and requires a combination of 2 antibiotics.^[12]^ Pitsouni et al showed that azithromycin has the same therapeutic effect as erythromycin with fewer side effects.^[13]^ We adjusted the use of erythromycin and azithromycin in combination with clinical symptoms and cerebrospinal fluid reexamination results. The treatment lasted for 5 weeks. The current treatment time for this type of infant meningitis ranges from to 3 to 7 weeks (Table 1).

In conclusion, neonatal Ureaplasma meningitis may be more common than previously suspected. The clinical manifestations were not obvious and were similar to those of neonatal meningitis caused by other bacteria. When conventional treatments and conventional pathogenic tests are negative, mNGS is a better choice for timely and accurate pathogen identification. For pathogens detected using mNGS, PCR can be used for further confirmation to provide more accurate clinical information.

## Acknowledgments

We thank the patient and his parents for permitting us to use the data and the Zhejiang Health and Science Technological Plan Program (2020KY279 & 2021KY1049 & 2021KY323).

## Author contributions

**Conceptualization:** Lyn Qin, Yan-hong Li.

**Data curation:** Xue-jie Cao, Xiao-jun Wang.

**Validation:** Ren-ping Mao, Hai-yin Yang, Li Li.

**Writing –original draft:** Yan-hong Li, Xue-jie Cao.

**Writing – review & editing:** Lyn Qin.
